# Factors influencing the detection and occupancy of little brown bats (*Myotis lucifugus*)

**DOI:** 10.1002/ece3.10916

**Published:** 2024-01-31

**Authors:** Tara C. Hohoff, Jill L. Deppe

**Affiliations:** ^1^ Department of Biological Sciences Eastern Illinois University Charleston Illinois USA; ^2^ Present address: National Audubon Society Washington DC USA

**Keywords:** acoustics, detection, false‐positive, mist netting, *Myotis lucifugus*, occupancy modeling

## Abstract

Using acoustics to survey for bats has increased as the need for data on increasingly rare species has also increased. We set out to better understand the difference between mist netting and acoustic detection probabilities between these two methods for the little brown bat (*Myotis lucifugus*), a species highly impacted by white‐nose syndrome and currently considered for federal listing in the United States. We also analyzed occupancy relationships with local and landcover variables. We surveyed 15 sites using mist netting paired with an acoustic recorder for multiple nights to estimate detection probability of this species. We also deployed acoustic recorders at another 73 sites. We found that detection rates for mist netting were very low but increased with day of year and decreased from proximity to water. Acoustic surveys had higher detection rates, but there was an approximately 30% likelihood of false‐positive detections. At the mean distance to water and day of year, acoustic surveys had a detection rate 55 times higher than mist netting. There were not significant factors influencing occupancy of little brown bats, only a slight positive relationship between forested largest patch, landscape patch richness and forest basal area. Given the declines in little brown bat populations since white‐nose syndrome, it is even more critical that we consider the very low detection rate of mist netting compared to acoustic surveys.

## Graphical abstract

The little brown bat has low detection estimates for mist netting compared to acoustic surveys. Higher survey effort recommended for this species using capture techniques.

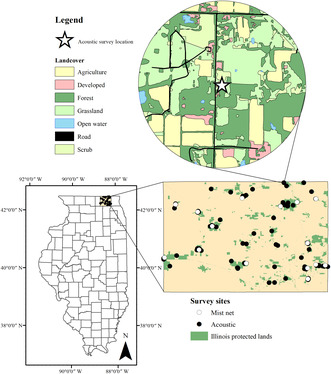

## 1 INTRODUCTION

When developing best practices for conservation, it is important to be able to conduct effective, precise surveys and properly interpret the results. For bats, survey methods have evolved from simple mist net setups (Greenhall & Paradiso, 1968) to complex arrays of acoustic microphones (Corcoran & Weller, 2018). Our capacity for analysis of data has also advanced from straightforward single species and season models to intricate models capable of community level analyses (MacKenzie et al., 2017). In addition to how we collect and analyze data, it is also important to consider the context of our research and interpret how results may differ within a species range. This is especially true for species that range across multiple ecological regions, such as the little brown bat (*Myotis lucifugus*; Fenton & Barclay, 1980).

The little brown bat has been historically common throughout its range up until the onset of white‐nose syndrome (*Pseudogymnoascus destructans*), which was confirmed in Illinois in 2013 (Kath, 2013). This species has adapted to frequently roosting in human structures such as barns and attics (Fenton & Barclay, 1980). Little brown bats migrate in the fall to caves to spend the winter months, where their survival is dependent on mitigating the impacts of frequent arousal from white nose syndrome (Auteri & Knowles, 2020). Little brown bats have experienced devastating population declines from this fungus (Frick et al., 2010), prompting a review of the species for protection through the United States Fish and Wildlife Service (https://ecos.fws.gov/ecp/species/9051). Given the current interest in little brown bats, it is important that we understand how to effectively survey this species with available technology and understand habitat selection relationships. We selected little brown bats for our analysis because they were frequently captured in the mist nets compared to other species at the time of survey, right at the time of confirmation of white‐nose syndrome in Illinois, meeting the minimum threshold for capture to estimate detection probability.

A combination of multiple methods for observational surveys is widely used throughout taxa to account for survey biases (Celis‐Murillo et al., 2009; Clare et al., 2017; Guzy et al., 2014; Nichols et al., 2008). While acoustic recorders can passively record vast amounts of data with minimal field effort, these files can be difficult to use for analysis due to inability to estimate populations and difficulty with some species identification (Lemen et al., 2015; Nocera et al., 2019; Russo et al., 2017). Mist netting requires greater survey effort in the field by a permitted biologist and typically yields lower detection of species (Murray et al., 1999; O'Farrell & Gannon, 1999). This technique is most effective in forested corridors that bats use to commute between roost and foraging habitat (Kunz & Kurta, 1998), limiting ability to survey different habitat types, including agricultural fields, grasslands and residential or developed areas. In order to provide data for multiple land cover types necessary for species habitat modeling, acoustic surveys are necessary. Although mist netting and acoustics are commonly used to survey bats, detection rates need to be regularly assessed as populations are in flux and available software and technology is constantly changing. The development of false‐positive occupancy modeling (Miller et al., 2011) lends itself well to the survey options available for bats (Clement et al., 2014). Mist netting provides a certain detection method, fitting the typical occupancy modeling framework (MacKenzie et al., 2002). While acoustics can provide additional data in land cover types where mist netting is less effective, with uncertain (possible false‐positive) species identification.

We used both mist‐netting and acoustic surveys to estimate the detection probability for the little brown bat, a species once common throughout its range but currently under review for federal protection. In addition to investigating factors influencing detection of this species, we also considered how local and landscape level metrics may impact occupancy. Little brown bats have been associated with edge habitat (Nelson & Gillam, 2017), urban environments (Coleman & Barclay, 2011), and open water (Bergeson et al., 2013). In our study we used the paired survey design in habitats where mist netting is most effective—forested corridors typically near water. With additional acoustic recorders in other types of land cover such as grassland, residential, and agricultural fields, to comprehensively survey the available landscape for occupancy analysis. We hypothesized that date would influence detection as maternal condition changes through the active season and young become volant (Deeley et al., 2022; Fenton & Barclay, 1980) increasing the number of individuals in the population for detection (MacKenzie et al., 2017). This was also a consideration for detection due to bats using waterways as flight corridors and foraging on aquatic insects (Hagen & Sabo, 2011). Since vegetation can minimize acoustic detection range (Weller & Zabel, 2002), we considered that density of trees at the survey location might have an interactive effect with survey method. We also included survey night in our analysis as these factors have been proposed to impact detection (Kaiser & O'Keefe, 2015; La Val, 1970). Our main goal is to determine detection probability of the two survey methods with secondary interest in other factors that influence detection and occupancy of the little brown bat.

## 2 METHODS

### 2.1 Study area

**FIGURE 1:**
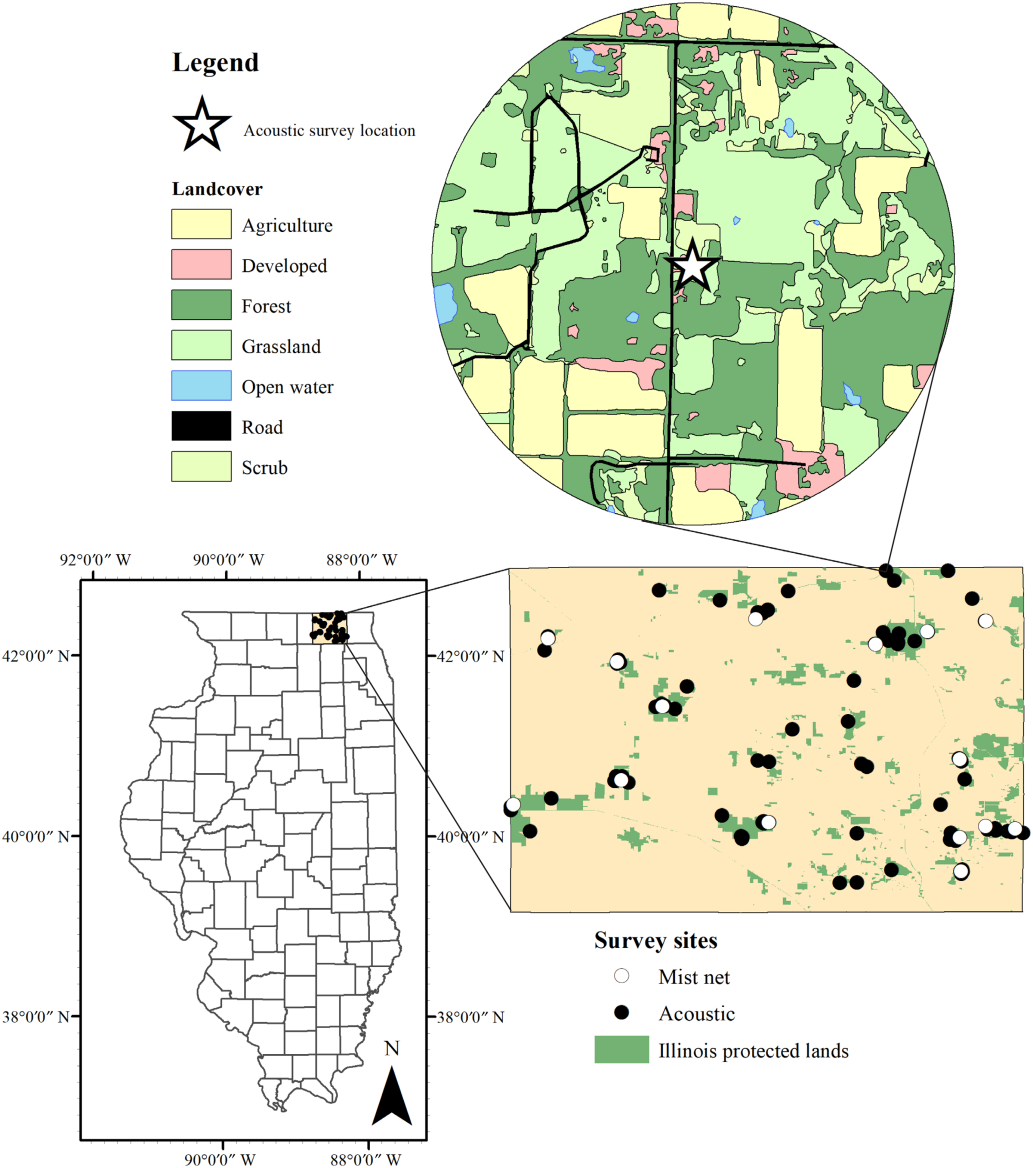
Map of McHenry County, Illinois, with acoustic and mist netting survey locations and magnified view of 1 km buffer to highlight the details of the land cover map for landscape level analysis.

We sampled for bats in McHenry County in northeastern Illinois, USA during the summers of 2013 and 2014 (Figure 1). McHenry County has a heterogeneous landscape providing a diversity of survey locations including developed, agriculture, grassland/prairies, and forested sites. This was crucial to our study design, as we wanted to avoid surveying in a single landcover type to understand how bats utilize habitat across a Midwestern landscape. All mist netting occurred on McHenry Country Conservation District property, most sites containing bottomland forest and water features. We surveyed at 14 unique parks with a mean size of 360 hectares, the smallest being 4 hectares and the largest 1375 hectares. Eight species of bats have been recorded in McHenry County: the big brown bat (*Eptesicus fuscus*), evening bat (*Nycticeius humeralis*), Eastern red bat (*Lasiurus borealis*), hoary bat (*Lasiurus cinereus*), little brown bat, northern long‐eared bat (*Myotis septentrionalis*), silver‐haired bat (*Lasionycteris noctivagans*), and tri‐colored bat (*Perimyotis subflavus*; Feldhammer et al., 2015).

### 2.2 Data collection and acoustic analysis

We conducted mist net surveys on McHenry County Conservation District property, mostly along forested water bodies, trails or roads. We used 38 mm‐mesh mist nets (Avinet, Dryden, NY), that were 6 m or 9 m long, depending on the width of the flight corridor, stream or pond edge following 2013 United States Fish and Wildlife guidelines for Indiana bat surveys. We resampled any sites in which there were less than 3 survey hours. In 2013, we surveyed 15 sites for two nights each between 10 June and 8 August and we resurveyed five of the 15 sites for five nights in 2014 between 3 June and 13 August plus one additional new site for one night.

We deployed SM2BAT+ recorders (Wildlife Acoustics, Maynard, MA) for two nights within approved weather guidelines (USFWS, 2023; no precipitation or high winds and temperature above 10°C) at 88 unique sites, including the 16 mist net sites. At mist net sites, we stationed the recorder within the same flight corridor as the mist nets, but far enough away to avoid detecting bats in the nets. The other 72 sites were dispersed throughout the county in agricultural fields, developed areas (e.g., residential backyards), forests, wetlands, and grasslands using one recorder for two nights. We recorded throughout the night as recommended by USFWS for acoustic surveys (2013). We utilized Kaleidoscope 5.1.9 (Wildlife Acoustics, Maynard, MA, USA), to process acoustic files to species with a minimum number of five call pulses (Britzke et al., 2011; Lemen et al., 2015) using the bats of North America 5.1.0 classifier on the “0 Balanced (Neutral)” setting. We allowed the program to only classify little brown and northern long‐eared bats (*Myotis septentrionalis*) within the *Myotis* genus, since we did not capture any Indiana bats (*Myotis sodalis*) and previous surveys have not documented the species in McHenry County (Hofmann, 2008). We considered a site occupied for that night if the maximum likelihood estimation (MLE) *p*‐value calculated by kaleidoscope for little brown bats was ≤.05.

### 2.3 Local and landscape variables

At each acoustic recorder location, we used a modified point‐quarter method to estimate tree density (Gehrt & Chelsvig, 2003) in forested locations to obtain mean distance to tree, which was then used to calculate tree density (1/mean plant distance^2^). We also measured the diameter at breast height (DBH) of the nearest tree in each quadrant for an average DBH. We used the 2014 National Agriculture Imagery Program (NAIP) imagery (U.S. Department of Agriculture, 2014) at 1 m resolution in ArcMap 10.2 to digitize the surrounding 1 km land cover into polygons including: developed, forest, agriculture, grassland, scrub, wetland and open water. We exported each buffer as a tiff image for import into Fragstats Version 4.2 (McGarigal et al., 2015) for measurement of landscape‐ and class‐level metrics. We used the United States Geological Survey National Hydrography Dataset (2018) to calculate the distance to nearest water and omitted ephemeral and intermittent streams since many of these are dry during the summer survey season.

### 2.4 Modeling and analysis

We fit numerical values into an unmarked frame using the package Unmarked 1.2.5 (Fiske & Chandler, 2011) in R Studio 2023.03.0 (RStudio: Integrated Development for R. RStudio, Inc., Boston, MA) using R version 4.3.0. We used the occuFP function to identify mist netting as our certain detections and nights with acoustic detections with MLE ≤0.05 as uncertain detections for calculation of false‐positive rate. We first developed detection models based on our hypotheses including method (acoustic vs. mist netting), day of year, year, survey night, mean tree density and distance to water. We developed 10 detection models and used Akaike's Information Criterion adjusted for small sample sizes (AICc) and Akaike model weights (*w*) to identify the best fitting model (Burnham & Anderson, 2002). We created a correlation matrix and used the package usdm (Naimi et al., 2014) to calculate the variance inflation factor (VIF) for our set of local and landscape occupancy covariates to detect multicollinearity among variables. We then built 16 models to estimate occupancy (Ψ) without combining variables into the same models with a VIF greater than 3 or correlation of greater than 0.5. We used the previously identified top detection covariates to build the occupancy models. Due to model uncertainty for occupancy, we used model averaged estimates using models within 2 ΔAICc, focusing on covariates represented in an equal number of models (Burnham & Anderson, 2002).

## 3 RESULTS

We captured 59 little brown bats at four of the 16 mist net sites for a naïve occupancy of 0.25, with all of the captures occurring on 11 of 56 survey nights. We deployed acoustic recorders in 29 forested sites, 32 grasslands, 13 developed/residential sites, 10 agricultural fields and 4 wetlands. The acoustic recorders detected little brown bats on 100 of 220 survey nights at 48 of 88 sites for a naïve occupancy of 0.55.

**FIGURE 2:**
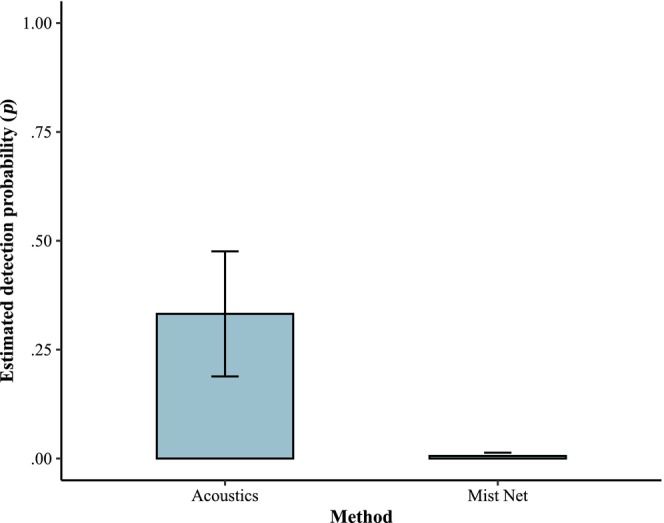
Estimated detection probability (*p*) for little brown bats at the mean day of year (189) and the distance to water (205 m) using false‐positive occupancy modeling for mist netting and acoustic surveys.

**FIGURE 3:**
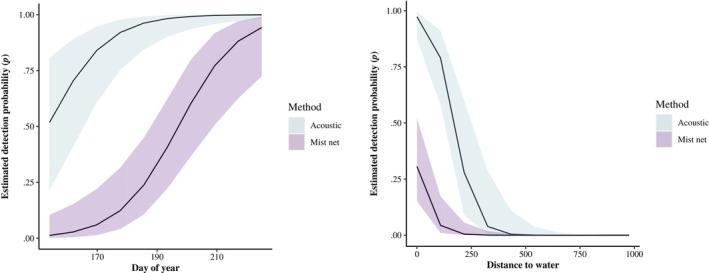
Estimated detection probability (*p*) based on the day of year at 0 m distance to the water and detection probability as related to the distance to water on the 210th day of the year.

Of the 10 detection models, the top model (*w* = 0.997) included method, survey date, and distance to water (Table 1). The method and date model was second (ΔAICc = 13.33, *w* = 0.001), and the method only model was ranked seventh (ΔAICc = 20.74, *w* = 0.001). The null model was ranked last with a ΔAICc of 50.74. At the mean survey date (189) and mean distance to water (205 m), the estimated detection probability for mist netting was 0.006 ± 0.007 and 0.332 ± 0.144 for acoustics (Figure 2). The relationship between detection and distance to water was negative, with higher detection closer to water and positive with day of year (Figure 3). Around mid‐July is when mist netting detection surpasses 0.50 at a 0 m distance to water. The estimated detection probability at the mean survey date (189) at 0 m from water is 0.31, while at 100 m away, the detection drops to 0.04. The false‐positivity rate was estimated at 30.8 ± 0.05%.

**TABLE 1:** Models for detection probability of the little brown bat using mist netting and acoustic survey techniques in a multi‐method occupancy modeling framework.

Model	*K*	AICc	ΔAICc	*w*	LL
*p*(Method + date + Water)	7	378.64	0.00	0.996898	−181.62
*p*(Method + date)	6	391.97	13.33	0.001271	−189.47
*p*(Method × Density + date)	8	392.49	13.85	0.000982	−187.33
*p*(Method + Water)	6	394.67	16.03	0.000330	−190.82
*p*(Method × Water)	7	394.94	16.30	0.000288	−189.77
*p*(Method × Night + date)	8	395.96	17.32	0.000173	−189.07
*p*(Method)	5	399.38	20.74	0.000031	−194.32
*p*(Method + Year)	6	400.46	21.82	0.000018	−193.71
*p*(Method × Night)	7	402.71	24.07	0.000006	−193.65
*p*(Method × Density)	7	403.81	25.17	0.000003	−194.21
*p*(.)	4	429.39	50.74	0.000000	−210.45

*Note*: We report the number of parameters (*K*), Akaike's Information Criteria adjusted for small sample sizes (AICc), the difference in AICc from the most parsimonious model (ΔAICc), model weight (*w*), and log‐likelihood (LL) estimates.

**FIGURE 4:**
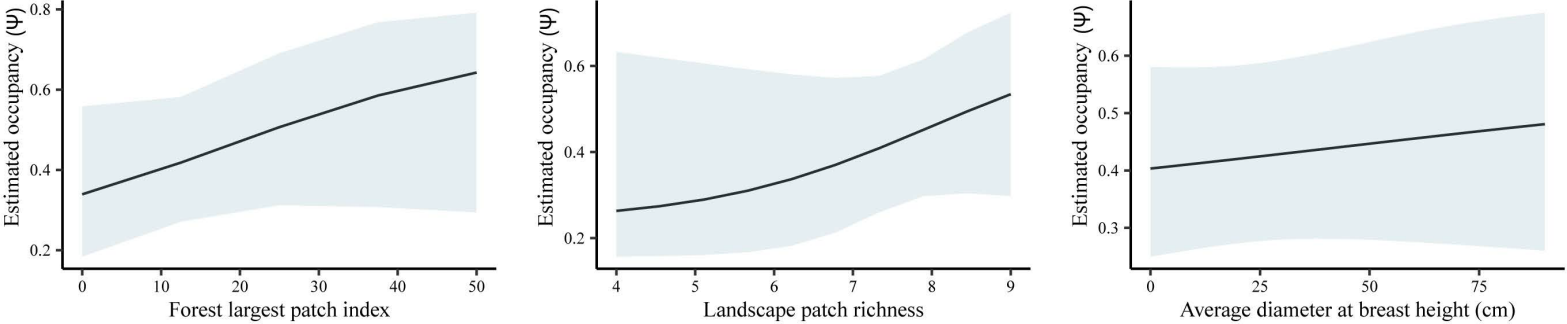
Model‐averaged estimated occupancy for the top three covariates using the models within 2 ΔAICc of the top model for little brown bats.

In the full candidate set of models including detection and occupancy, there were nine models within 2 ΔAICc, including the top detection only model, *p*(Method + date + Water), which ranked third (Table 2). The top model included only forested largest patch index and the second model included forested largest patch index and landscape patch richness. The model‐averaged estimates for occupancy using the models within 2 ΔAICc identified only slight positive relationships with largest forested patch, landscape patch richness, and average DBH (Figure 4).

**TABLE 2:** Occupancy and detection models, including the number of parameters (*K*), Akaike's information criteria adjusted for small sample sizes (AICc), the difference in AICc from the most parsimonious model (ΔAICc), model weight (*w*), and log‐likelihood (LL) estimates.

Models	*K*	AICc	ΔAICc	*w*	LL
*p*(Method + date + water), Ψ(~ FOR_LPI)	8	378.19	0.00	0.137238	−180.18
*p*(Method + date + water), Ψ(~ FOR_LPI + Lndscp_PR)	9	378.61	0.43	0.110959	−179.15
*p*(Method + date + water)	7	378.64	0.46	0.109304	−181.62
*p*(Method + date + water), Ψ(~ Lndscp_PR)	8	379.16	0.98	0.084166	−180.67
*p*(Method + date + water), Ψ(~ FOR_AREA)	8	379.92	1.73	0.057738	−181.05
*p*(Method + date + water), Ψ(~ GSS_NP)	8	379.92	1.74	0.057601	−181.05
*p*(Method + date + water), Ψ(~ DBH + Lndscp_PR)	9	380.06	1.87	0.053831	−179.88
*p*(Method + date + water), Ψ(~ DBH)	8	380.06	1.87	0.053807	−181.12
*p*(Method + date + water), Ψ(~ FOR_LPI + DBH)	9	380.14	1.95	0.051684	−179.92
*p*(Method + date + water), Ψ(~ FOR_NP)	8	380.21	2.02	0.049970	−181.19
*p*(Method + date + water), Ψ(~ AG_AREA)	8	380.24	2.05	0.049173	−181.21
*p*(Method + date + water), Ψ(~ FOR_AREA + Lndscp_PR)	9	380.80	2.61	0.037229	−180.24
*p*(Method + date + water), Ψ(~ DV_AREA)	8	380.97	2.78	0.034205	−181.57
*p*(Method + date + water), Ψ(~ GSS_NP + Lndscp_PR)	9	380.99	2.80	0.033792	−180.34
*p*(Method + date + water), Ψ(~ GSS_AREA)	8	381.06	2.87	0.032610	−181.62
*p*(Method + date + water), Ψ(~ Water)	8	381.62	3.43	0.024678	−181.90
*p*(Method + date + water), Ψ(~ FOR_AREA + DBH)	9	381.88	3.69	0.021675	−180.79
*p*(Method + date)	6	391.97	13.78	0.000139	−189.47
*p*(Method × density + date)	8	392.49	14.30	0.000108	−187.33
*p*(Method + water)	6	394.67	16.48	0.000036	−190.82
*p*(Method × water)	7	394.94	16.76	0.000032	−189.77
*p*(Method × night + date)	8	395.96	17.78	0.000019	−189.07
*p*(Method)	5	399.38	21.19	0.000003	−194.32
*p*(Method + year)	6	400.46	22.27	0.000002	−193.71
*p*(Method × night)	7	402.71	24.52	0.000001	−193.65
*p*(Method × density)	7	403.81	25.62	0.000000	−194.21
*p*(.), Ψ(.)	4	429.39	51.20	0.000000	−210.45

## 4 DISCUSSION

The use of acoustic data for bats is critical to understanding bat distribution patterns, due to the limitations of mist netting to provide adequate capture data with reasonable survey effort for statistical analysis (Weller & Lee, 2007) and to survey outside of forested corridors. The paired method survey design provides necessary data for effective calculation of detection probability and false‐positive detection rate. With little brown bats under review by the United States Fish and Wildlife Service for federal protection, it is imperative we understand best survey practices to monitor these populations. Our detection probability estimates highlight the low success rate for capture of little brown bats, a species that during the time of survey was still relatively abundant before the onset of white‐nose syndrome. This corroborates similar studies conducted in the western United States confirming low detection for this species (Rodhouse et al., 2012; Weller, 2008). Given population trends since the time of survey, current detection rates are probably much lower since abundance impacts detection rate (MacKenzie et al., 2017) and we know this species has experienced significant declines in areas impacted by white‐nose syndrome (Deeley et al., 2021; Frick et al., 2010). According to simulation models by MacKenzie et al. (2002) for accurate estimates of occupancy, detection probability needs to be >0.3 for only two site visits where occupancy is at least >0.7. Our estimates for detection, especially for mist netting were low, as well as occupancy estimates, with most values less than 0.6. The calculation of these detection rates would not be possible from mist‐netting in the Midwest post white‐nose syndrome as capture rates of little brown bats are so low (Pettit & O'Keefe, 2017).

The negative relationship with distance to water and positive relationship with day of year for detection suggests these should be factors in calculating effective survey effort. We suggest that there may need to be greater survey effort at sites further from water due to the decreasing detection rate as distance to water increases if little brown bats are added to current United States Fish and Wildlife guidance (USFWS, 2023). Site selection for acoustics should prioritize locations near water or may require longer survey effort. All of our survey sites were within 1 km of water so additional data on greater distances away would be beneficial. With the United States Fish and Wildlife guidelines identifying the survey season as 15 May to 15 August (USFWS, 2023), there may be benefits to increasing levels of survey effort early in the field season. Detection probability increasing later in the summer is likely due to the young of the year taking flight (Kaiser & O'Keefe, 2015; Pauli et al., 2017)., increasing abundance and likelihood of detection (Clement et al., 2014) or possibly changes in foraging patterns through maternal progress (Deeley et al., 2022).

Our estimated false‐positive detection rate of 30% is higher than the 14% reported by Clement et al. (2014). While they used manual review to verify detections, we chose to only use auto‐identification output for our estimates as many organizations do not have access to trained biologists and manual review introduces additional bias to be estimated (Farmer et al., 2012; Fritsch & Bruckner, 2014), including variability among reviewers of bat echolocation files.

The lack of strong associations for occupancy supports the little brown bat being a habitat generalist, which may explain why there are conflicting studies on habitat preference for this species. For example, being identified as abundant (Coleman & Barclay, 2011) and not abundant in urban areas (Kurta & Teramino, 1992). This species has adaptive roosting strategies compared to other *Myotis* which allows more flexibility in habitat preference (Bergeson et al., 2015). Given the vast species range of the little brown bat, it makes sense that it would be capable of using different habitat types depending on availability of resources (Fenton & Barclay, 1980). We recommend that these estimates of detection and occupancy are considered when determining survey effort required for this species. We also suggest that research continues to use a paired method design to continue to explore how detection rates change with species, abundance, region, equipment and software used (Adams et al., 2012; Lemen et al., 2015).

## AUTHOR CONTRIBUTIONS

**Tara C. Hohoff:** Data curation (lead); formal analysis (lead); writing – original draft (lead). **Jill L. Deppe:** Conceptualization (equal); funding acquisition (lead); methodology (equal).

## CONFLICT OF INTEREST STATEMENT

There are no competing interests to disclose regarding this manuscript.

## Data Availability

Data and code are available via the Illinois Data Bank: https://doi.org/10.13012/B2IDB‐0365076_V1.
